# Anxiety of mothers of newborns with congenital malformations in the pre- and postnatal periods*

**DOI:** 10.1590/1518-8345.2482.3080

**Published:** 2018-11-14

**Authors:** Fabíola Chaves Fontoura, Maria Vera Lúcia Moreira Leitão Cardoso, Sofia Esmeraldo Rodrigues, Paulo César de Almeida, Liliane Brandão Carvalho

**Affiliations:** 1 Faculdade de Enfermagem Nova Esperança, Mossoró, RN, Brazil.; 2 Universidade Federal do Ceará, Faculdade de Farmácia, Odontologia e Enfermagem, Fortaleza, CE, Brazil.; 3 Hospital Geral de Fortaleza, Unidade Neonatal, Fortaleza, CE, Brazil.; 4 Universidade Estadual do Ceará, Centro de Ciências da Saúde, Fortaleza, CE, Brazil.; 5 Universidade de Fortaleza, Departamento de Psicologia, Fortaleza, CE, Brazil.

**Keywords:** Congenital Abnormalities, Anxiety, Mothers, Newborn, Intensive Care Units, Prenatal Care

## Abstract

**Objective:**

to analyze the anxiety level of the mothers of newborns with congenital malformations who were diagnosed in prenatal and postnatal care.

**Methods:**

a cross-sectional study with 115 mothers of 117 newborns with congenital malformation admitted to three neonatal units. A questionnaire containing maternal and neonatal variables was used, as well as the Trait-State Anxiety Inventory. Data were analyzed by Student’s t-test and Kolmogorov-Sminorv test. The anxiety level was categorized as low (percentile <25), moderate (25-75) and high (> 75), with a significance level of 5%.

**Results:**

most mothers had moderate levels of anxiety. Regarding the diagnosis of the malformation, 57% received the news in the prenatal and 43% in the postnatal period. The anxiety level of those who received the prenatal diagnosis was lower than those who received in the postnatal period, evaluated by the Trait Anxiety Inventory (p = 0.026).

**Conclusion:**

mothers of newborns with malformations presented moderate anxiety, and this was higher when the diagnosis was given in the postnatal period. The use of the Trait-State Anxiety Inventory can provide guidance to other studies and to clinical practice.

## Introduction

Gestation is a phase of transition in the life of the woman in which, from the moment of fertilization until birth, the pregnant woman and the baby go through unique psychological, physiological and social experiences, causing the woman to have doubts and expectations in relation to the new being that is yet to be born and to the role she will have to assume^1^ ^-^ ^2^.

However, receiving the news that your baby has congenital malformation is a difficult and unique moment, in which the mother experiences a period of mourning, when the idealized baby ceases to exist^3^. When the malformation is incompatible with life, the situation can lead to great suffering and implications for the pregnant woman, her partner and other relatives, bringing feelings of frustration, guilt, disability and loss, crises in the family system and social isolation^4^. It can also lead to the feeling of anxiety, which runs through time. Still, most malformed babies are referred to the neonatal unit, causing momentary separation of the mother-child binomial.

In Brazil, statistical indices show that congenital malformations constitute the second cause of neonatal mortality, accounting for 22.8% of all deaths. The Southeastern and North regions present the highest proportion of death records for this cause, accounting for 35.9% and 24.5%, respectively^5^. A study about the prevalence of malformations in São Paulo concluded that early diagnosis of malformations is important to reduce early neonatal morbidity and mortality and to improve quality of life and survival rates^6^. 

 Being able to deal with the prognosis of congenital malformation depends on several factors, namely the emotional structure of the couple and the family, as well as the specialized health care and the multiprofessional care provided^7^. For the family, especially the mother, dealing with this situation can trigger some level of anxiety.

Anxiety is a common mental disorder in the pregnancy-puerperal cycle as a result of changes and psychological and social adaptations in women’s lives. It is the most common type of disorder in this population and usually occurs in all ages^8^. Mental disorders in the gestational period are quite prevalent and common, and multiple risk factors are involved in their genesis. However, they are still underdiagnosed^9^. During the postpartum period, researchers report that 11% of mothers of healthy newborn develop symptoms of depression within 72 hours after birth^10^ and that after six weeks they also presented symptoms of traumatic stress and/or anxiety^11^. In the postpartum period, there is an increased risk of suffering, especially among mothers of newborns with confirmed malformations, who will stay hospitalized after birth. Therefore, the early identification of psychological distress in this period and the referral to mental health care providers within the obstetric environment are essential^12^. 

As an ally in the evaluation of anxiety and the behavior of pregnant women after receiving the diagnosis of congenital malformation, a study carried out in a hospital environment used tools, more specifically scales, that were appropriately validated and multidimensional, adapted to the Brazilian population, in an attempt to understand the coping strategies, as well as the thoughts and actions adopted in the face of a particularly stressful event^13^. 

There has been scarcity of studies that evaluate the impact of the news of congenital malformation in the different periods of gestation^1^, and this encouraged the development of the present study, which shows to be relevant due to its great value in investigating the anxiety level experienced by the mothers during this transition phase of their lives. Thus, based on the results, health professionals, especially nurses, can encourage health promotion actions according to women’s needs, contributing to the strengthening of emotions, demystifying fantasies, creating bonds and fortifying the relationship between family and the newborn. 

Nursing, as a profession, like the other health areas, uses technologies to implement assistance to its clients and to promote autonomy and independence, whether in closed institutions, such as hospitals, for health education or in any other environment^14^. 

In this context, the research questions are: What is the anxiety level of the mothers of newborns with malformations hospitalized in the Neonatal Unit of hospital institutions, according to the Trait-State Anxiety Inventory (STAI)? Is there a difference in maternal anxiety levels when the malformed child is diagnosed? Thus, the objective was to analyze the anxiety level of the mothers of newborns with congenital malformations who received the diagnosis in the prenatal or postnatal period. 

## Method

This was a quantitative, cross-sectional and comparative study carried out in three tertiary-level hospitals in Fortaleza-CE, in a neonatal admission unit and rooming-in, a reference in neonatal care to the rural and urban population of the state. The study population was composed of puerperal women whose children had congenital malformations diagnosed during the prenatal or postnatal period and were hospitalized at the neonatal unit during the period of data collection. The sample was selected by convenience, in which the neonates were initially captured in the neonatal units and, later, the mothers were located to apply the research eligibility criteria, totalizing a sample with 115 mothers during the period from May 1, 2014 to April 30, 2015. 

Inclusion criteria were mothers of newborns with any type of congenital malformation; malformation having been diagnosed during prenatal care, during the neonatal evaluation in the delivery room or during the first 7 days of life, during which the newborn was still hospitalized in the neonatal unit; mothers who were physically and psychologically able to answer the research questionnaires; mothers who were accompanying the newborn during the period of hospitalization in the neonatal unit; and newborns who had not died prior to application of the instrument. Mothers with psychiatric antecedents, who presented signs of delusions and/or hallucinations, carrying the Human Immunodeficiency Virus, with impaired hearing, presenting clinical complications in the puerperium, using psychotropic medications and who were discharged before the confirmation of the diagnosis of congenital malformation at birth. 

The data were collected by the researcher during the daytime period, as it was the moment when the family was more available and were visiting the newborn in the units, which favored making the invitation for participation. The instruments were applied in the first week after birth (up to 7 days), this period being the most favorable period for this collection because the newborn was still hospitalized in the neonatal unit^15^.

Daily telephone contacts were made with the nurses of the units surveyed, as well as periodic visits to the institutions for finding these newborns and their mothers. Subsequently, the instruments used in the study were applied: a questionnaire containing sociodemographic, maternal and neonatal variables; and the Trait-State Anxiety Inventory (STAI).

The STAI is a self-assessment questionnaire for adults^16^ adapted to Portuguese language^17^. It is composed of two scales, designed to measure two distinct concepts of anxiety: anxiety state (STAI-State), which evaluates how the individual feels at the moment of the interview, and the anxiety trait (STAI-Trait), which evaluates how the person usually feels^18^. The STAI scale cutoff points were defined from percentiles (25 and 75), since the author of the scale did not define the intervals and cutoff points for determining the different anxiety levels. Therefore, low anxiety corresponded to the percentile <25; moderate, from 25 to 75; and high, to the percentile > 75.

The data were processed in the Statistical Package for the Social Sciences, version 20.0, license number 10101131007. The descriptive statistics mean, standard deviation and percentiles of the STAI-Trait and STAI-State scales were used. The normality of the scores of these scales was verified by the Kolmogorov-Sminorv test. The anxiety levels of the mothers who received the diagnosis during prenatal care and those who received it in the postnatal period were compared by Student’s t for independent data. Statistical analyzes were considered statistically significant at p <0.05.

To formalize the inclusion of the mothers in the study, after having accepted to participate in the research, they were requested to sign of the Informed Consent Form. If the mother was less than 18 years old, her legal guardian was contacted to sign of the form. The research was approved under opinion 618.031 of April 16, 2014, and the ethical principles of resolution 466/12 of the National Council of Ethics in Health of Brazil for research involving human beings were respected. 

## Results

Among the 115 mothers studied, 50% were aged between 19 and 29 years, 47% were from Fortaleza, 50% lived in stable union with their partners, 52% had studied for 6 to 10 years, 93% declared to be browns, 41% were primigravida, 83% had undergone cesarean delivery and 4% had used licit and/or illicit drugs during gestation. There was variation regarding anxiety, as detected by the STAI, according to Table 1.

**Table 1:** Anxiety levels of mothers of newborns with congenital malformations hospitalized in neonatal units according to the STAI* scale. Fortaleza, CE, Brazil, 2015

Percentile Intervals	n^†^	%	Mean (SD)^‡^	
STAI - Trait^§^			39.06 (9.16)	
Percentile <25 (low anxiety)	31	27.0		
Percentile 25-75 (moderate anxiety)	62	53.9		
Percentile> 75 (high anxiety)	22	19.1		
STAI - State^||^			46.79 (8.77)	
Percentile <25 (low anxiety)	33	28.7		
Percentile 25-75 (moderate anxiety)	54	47.0		
Percentile> 75 (high anxiety)	28	24.3		

*STAI - State-Trait Anxiety Inventory; †n - absolute number; ‡SD - standard deviation; §STAI-Trait - Trait Anxiety Inventory; ||STAI-State - State Anxiety Inventory

The majority of the mothers presented total scores inserted in the percentile range between 25 and 75, characterizing moderate levels of anxiety. 

**Figure 1:**
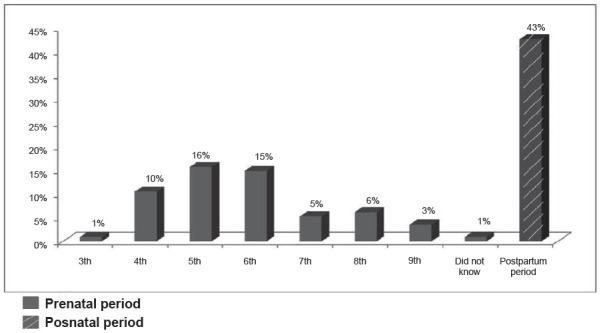
Period in which the investigated mothers received the diagnosis of congenital malformation. Fortaleza, CE, Brazil, 2015

Of the 99% mothers who had performed prenatal care, 57% had the news of the congenital malformation of their children during this period, and it was more prevalent in the fifth and sixth months; 43% only received the diagnosis in the postnatal period (Figure 1).

**Figure 2:**
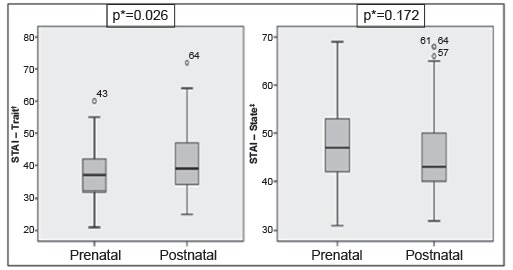
Comparison between the means (standard deviation) of the levels of maternal anxiety according to the period of diagnosis of the malformed child and the scales applied. Fortaleza, CE, Brazil, 2015

Figure 2 shows the comparison of the anxiety level among the mothers who received the diagnosis in the pre- and postnatal period.

*Student-t test (p <0.05); †STAI-Trait - Trait Anxiety Inventory; ‡STAI-State - State Anxiety Inventory

Before analyzing the means of the scales, we performed the Kolmogorov-Smirnov test, which indicated that they did not present normality: STAI-Trait (p <0.0001) and STAI-State (p = 0.001). Despite this, the Student’s t-test was used for this analysis, since the sample size was large enough (n = 115) to do so, according to the Central Limit Theorem^19^.

Only the means of the STAI-Trace showed statistically significant differences (p = 0.026), when the average anxiety level of those who received the diagnosis of congenital malformation in the prenatal period (37.4, SD = 7.034) was lower than that of mothers who received in the postnatal period (41,27, SD = 11,135). On the other hand, the averages of the STAI-State of the group of women who received the diagnosis of malformation in the prenatal period were higher (47.76, SD = 8.298) than women who were informed in the postnatal period (45.49, SD = 9.310), but without statistical significance (p = 0, 172).

As to the types of malformations of the newborns of the mothers composing this sample, these were grouped into the following categories: malformations of the musculoskeletal system (30.1%), central nervous system (20.4%), circulatory system (16.7%), cleft lip and/or palate (7.5%), eye, ear, face and neck (5.9), genitals (4.8%), other malformations of the digestive tract 4.3%), urinary tract (4.3%), chromosomal anomalies (2.7%), other malformations (2.7%) and those of the respiratory system (0.5).

## Discussion

In general, the results showed that the mothers of malformed newborns presented different anxiety levels during the pre- and postnatal periods. For parents, the news of a diagnosis of congenital anomaly in their child can trigger many reactions, which may vary according to, for example, the moment the diagnosis is revealed^20^.

Some studies state that admission to and hospitalization in the neonatal intensive care unit of preterm, critically ill, small for gestational age infants or with congenital malformation can be stressful events for parents. The technological apparatus of the unit, the appearance of the baby and the feeling of loss of paternal and maternal roles contribute to the level of stress among these parents. However, the major stressors for these are the following factors: pre- and postnatal experience; clinical diagnosis of the newborn; concerns about the recovery of the child; loss of maternal or paternal role; and being care providers^21^.

Experiencing hospitalization, separation, procedures and surgeries necessary for the child interferes with the emotional balance of the mothers and triggers high levels of stress and anxiety, followed by doubts and questions. It is up to health professionals to provide technical and emotional support to these mothers, which means promoting simple, clear humanized care, listening and dialogue in order to reduce stress, anxiety and inappropriate behavior^3^.

A systematic review of epidemiological studies that investigated the prevalence and association between maternal mental disorders and congenital malformations of the baby was carried out in a systematic review aimed at investigating the reactions experienced by mothers as a result of the diagnosis of congenital malformations in the child. The results showed that the prevalence of mental disorders differed widely from one study to another, with anxiety and psychic suffering being the most investigated disorders and with high frequency, both ranging from 13 to 60%, followed by depression, often between 13 and 27%, and high levels of stress (13%)^22^.

A Brazilian study, which evaluated the impact of the diagnosis of congenital malformation on the mental health of pregnant women undergoing prenatal care showed that all women of the sample had anxiety and 78% had depression, both of which were at low levels, with 38% and 62%, respectively. The news of the malformation in the first trimester showed association with anxiety. The number of pregnant women with signs of moderate anxiety (23%) also deserves attention^1^, which corroborates with the findings of the present study, in which 53.9% and 47% of the mothers investigated had moderate anxiety levels in both the IDATE-Trait and in the IDATE-State evaluation. Another research carried out in two cities in the interior of São Paulo state aimed at evaluating how maternal-fetal attachment is established, the coping modes, the clinical indicators and anxiety and depression in the prenatal phase in pregnant women who had received the diagnosis of the congenital malformation showed that the scores for anxiety were high. Among the pregnant women, three (13.7%) showed minimal anxiety; eight (36.4%) mild anxiety, two of whom also presented indicators for depression; another eight (36.4%), moderate anxiety, among which one of them also presented an indicator for depression; and three (13.6%) pregnant women had severe anxiety^23^. 

In another study, 45.5% of the mothers of malformed newborns with malformations and 18.2% of the mothers of normal infants presented clinical symptoms of anxiety-state. Regarding anxiety-trait, 36.4% of the mothers in the case group presented clinical symptoms, but the same result did not occur in mothers of neonates without malformations. As the authors also investigated associated depression symptoms, 22.7% of mothers of malformed infants and 4.5% of mothers of normal babies had indicative scores for both depression and anxiety symptoms^15^.

The fact that the mother is primiparous is also a strong indicator of association with anxiety, as shown in another Brazilian study that analyzed anxiety and maternal coping in the presence of a congenital anomaly, which identified association between maternal variables and emotional indicators. The results showed a strong association between the mother being primiparous and the minimum level of anxiety (p = 0.019), as well as the majority of the mothers received the news of the diagnosis of malformation before birth^24^.

The follow-up visits of infants diagnosed with malformations are also a triggering factor for psychological effects in pregnant women. A study presented mean scores of the STAI-State of mothers at 52.40 (SD = 9.08) in the first visit, 45.60 (SD = 8.40) in the second, 44.00 (SD = 7.55) in the third, and 38.00 (SD = 5.90) in the fourth visit, that is, the mothers’ anxiety in the first visit presented the highest mean score, since they did not know whether the child would be diagnosed with anomalies^25^. 

Anxiety is usually associated with other types of mental changes and/or feelings. Research conducted in Iran described anxiety and depression as associated disorders that were investigated in mothers during the postnatal period who had high-risk pregnancies. The prevalence of symptoms of depression and moderate anxiety was higher among women who had a high-risk pregnancy than those with normal pregnancies. However, anxiety was more prevalent than depression in both groups^26^.

When comparing the anxiety levels between the mothers who received the diagnosis of the malformation of the child in the prenatal period and those in the postnatal period, only the means of the STAI-Trait scale presented statistically significant differences (p = 0.026), in which the mean and the standard deviation of the scores that refer to the anxiety of those who received the diagnosis in the prenatal period were lower than the mothers who received it in the postnatal period. 

Diverging from such data, a study that analyzed the parents’ emotional reactions, including the mothers’ reactions, and the intensity of each emotion when the diagnosis of the congenital malformation is disclosed in the prenatal or postnatal period verified that the moment of the disclosure of the diagnosis (prenatal vs. postnatal) did not present a statistically significant association with the patterns of maternal emotional reactions, with p = 0.235 (chi-square of 1.41), and anxiety was included in it. By associating these moments with the intensity of the different emotions, statistical significance was found only in relation to anger and sadness. Only mothers who had received the diagnosis of malformation of their child during the prenatal period presented statistical significance for the feeling of anger, being more intense (p = 0.004), and for sadness (p = 0.044), than mothers who had received the diagnosis of congenital malformation after birth^27^.

A study that evaluated individual adjustment (psychopathological symptomatology and quality of life) and family impact (global and financial overload) of parents of children with a diagnosis of congenital malformation and the influence of the determinants of the child on the individual adjustment and parental overload found prevalence of 67.7% of the cases as having been diagnosed in prenatal care, with urological pathologies (33.9%) being the most frequent malformations^28^. The psychopathological symptomatology was verified by the Brief Symptom Inventory (BSI-18), consisting of 18 items, organized in three dimensions: anxiety, depression and somatization, and by a overall severity index. It was perceived, from univariate tests, significant effects on the three dimensions (ηp_2_ = 0.23, 0.09, 0.07, respectively) and on the overall severity index (ηp_2_=0.17), which showed that the mothers presented high scores of the symptoms.

One should be very cautious in talking to pregnant women and their companions about suspected and confirmed diagnoses of fetal malformation. In suspect cases, which refer to the possibility of the child having a malformation, as well as being subject to correction or not, the team may ensure whether disclosing this information is really necessary. Due to the possible negative impacts resulting from this news, especially when it refers to the relationship of maternal-fetal attachment and in the process of parenting, it may be wise to provide this information only when there is confirmation of the diagnosis^4^.

This research had some limitations, such as not following these mothers after hospital discharge, since the anxiety experienced at that time could decrease or increase over time due to possible morbidities caused by the malformation of the child or even deaths, in more severe cases or incompatible with life. In addition, we also had difficulty in finding the complete records of the diagnoses of the newborns. Due to the fact that the malformations are not of compulsory notification, in certain medical records it was not possible to obtain the complete information, so we need to seek help from professionals working in the sector for clarification.

As a suggestion for other studies, we suggest longitudinal follow-up of these mothers in order to contribute to a better adaptation to the experienced situation, as a way to minimize or prevent other mental disorders common to this population. 

## Conclusion 

The application of the STAI revealed that the mothers of newborns with malformations presented moderate anxiety. The news of the malformation of the child, received by the mothers in the postnatal period, triggered higher levels of anxiety than those who received it in the prenatal period, but this difference occurred only in the evaluation through the STAI-Trait scale. The use of technologies, such as the STAI, can provide guidelines for other studies and clinical practice.
